# Bridging the Communication Gap: Comparing Digital Tools and Traditional Methods in Post-Extraction Care Delivery

**DOI:** 10.3390/healthcare14121719

**Published:** 2026-06-15

**Authors:** Rajashekhara Bhari Sharanesha, Alwaleed Abushanan, Deepti Virupakshappa, Abdullah Bin Nabhan, Maram Alagla, Abdulhamid Al Ghwainem, Sara Alghamdi, Abdulaziz Fahad Alrubayyi, Majed Mohammed Kariri, Yousef Alkhaibari

**Affiliations:** 1 Department of Pediatric Dentistry, College of Dentistry, Prince Sattam Bin Abdulaziz University, Al-Kharj 11942, Saudi Arabia; a.abushanan@psau.edu.sa (A.A.); m.alagla@psau.edu.sa (M.A.); a.alghwainem@psau.edu.sa (A.A.G.); sar.alghamdi@psau.edu.sa (S.A.); 442050998@std.psau.edu.sa (A.F.A.); 442050400@std.psau.edu.sa (M.M.K.); 2 Department of Maxillofacial Surgery and Diagnostic Sciences, College of Dentistry, Prince Sattam Bin Abdulaziz University, Al-Kharj 11942, Saudi Arabia; d.virupakshappa@psau.edu.sa (D.V.); a.binnabhan@psau.edu.sa (A.B.N.); y.alkhaibari@psau.edu.sa (Y.A.)

**Keywords:** digital mode, leaflets, post-extraction care, QR codes, traditional methods, verbal instructions

## Abstract

**Highlights:**

- Digital tools, especially QR code-based videos, are widely perceived by dental students and patients as providing post-extraction care instructions after routine tooth extraction, compared with traditional methods.
- A cross-sectional study involving 200 dental students and 200 patients showing 67.5% of participants believe QR code-based techniques are better for enhancing patient-provider communication during post-extraction care.
- QR code-based instructions are highly preferred across various age groups, including elderly patients, indicating their potential to improve accessibility and understanding in post-extraction care.
- The support of combining communication strategies with traditional approaches in modern dental practices aims to enhance patient education and engagement in post-extraction care.

**Abstract:**

Background: Clear post-extraction sessions are vital for good patient outcomes. Traditional methods, such as verbal and printed information, have historically been prevalent; however, digital health care methods for analysis and communication are increasingly being adopted. Still, evidence comparing preferences for digital versus traditional instruction, especially for routine post-extraction care, remains limited. Objectives: This study aimed to evaluate preferences between digital methods (QR code-based videos) and traditional methods (verbal and printed leaflets) for delivering post-extraction care instructions among dental patients and students. It also assesses perceptions of communication quality, confidence, and patient interaction associated with each method. Methods: A cross-sectional study involved 200 dental students and 200 patients undergoing routine tooth extractions. The sample size was determined based on a 5% margin of error at a 95% confidence level for proportion estimation. Participants experienced all three instructional delivery methods—verbal, printed leaflet, and QR code-based video—for post-extraction care and completed validated questionnaires. The study assessed preferences for each delivery method, communication quality, confidence in following or providing instructions, ease of access, perceived usefulness, and impact on patient-provider interaction. Data were analyzed using descriptive statistics, the Mann–Whitney U test for group comparisons, and Spearman correlation for relationships among ordinal data. Results: Among all participants, 67.5% chose QR codes as the preferred method for improving communication (*p* < 0.001). Among dental students, 49% favored QR codes as the best method for postoperative instructions, and 50.5% indicated that QR codes boosted their confidence in providing instructions to patients. Preference for QR codes ranged from 43.5% to 65.5% across different aspects evaluated. Notably, among patients aged 60 years or older, 65.5% considered QR codes the most convenient for the elderly. Mann–Whitney U tests showed statistically significant differences between students and patients for ease of access (*p* = 0.009, rank biserial r = −0.143), video length appropriateness (*p* = 0.003, rank biserial r = −0.160), and the unlikelihood of missing instructions (*p* < 0.001, rank biserial r = −0.446). Conclusions: QR code-based video instructions were widely preferred over traditional methods by both dental students and patients for post-extraction care delivery. These findings support the integration of digital communication tools into post-extraction care protocols as a complement to traditional instruction delivery methods, though longitudinal studies assessing actual clinical outcomes are needed.

## 1. Introduction

Tooth Extraction is one of the most common dental procedures worldwide, and proper postoperative care and instruction are pivotal for preventing complications such as excessive bleeding, dry socket, infection, and delayed healing [1,2]. Post-extraction instructions typically include guidance on pain management, oral hygiene, activity restrictions, dietary limitations, and warning signs of complications that need professional care [3,4]. The clarity and accessibility of these instructions can significantly influence patient outcomes and satisfaction [5].

For many years, dentists have been providing post-extraction instructions through traditional methods like verbal explanations and printed materials, which were regarded as the standard practice [6,7,8]. These methods allow immediate clarification and personalized communication but are constrained by limited time, potential information overload, and reliance on patient memory [9,10]. Leaflets provide a tangible reference but have limitations, including literacy levels, language barriers, and lack of engagement [11,12]. Moreover, recent research shows that video instructions enhance patient understanding and satisfaction more than leaflets [10,13]. Healthcare-related mobile apps also show promise for increasing patient engagement and adherence to care [14].

Digital health technologies have progressed quickly, creating new ways to improve communication between patients and healthcare providers. Among these digital tools, quick response codes provide several advantages over traditional methods [15,16]. These enable easy, immediate access to several types of content, such as images, videos, and interactive materials, which can improve patients’ understanding and memory of post-extraction instructions [17,18]. These developments illustrate the growing trend of innovation in healthcare digitization and education, where both patients and students are becoming more accustomed to mobile technology [19,20]. However, most studies focus on specific patient groups or clinical situations, with limited direct comparisons between digital and traditional methods for post-extraction instructions, particularly in settings involving both healthcare trainees and patients [21,22].

Dental students represent the next generation of practitioners, and their comfort with digital communication may influence future adoption in clinical practice [8,23]. Understanding both students’ and patients’ preferences for instructional delivery methods can inform educational strategies and clinical protocols [24,25]. Furthermore, the acceptance of digital tools among different age groups, especially older adults, depends on their levels of technical literacy. This variability is a key factor influencing the practicality and potential for broad adoption [26,27,28].

However, gaps in research interest in digital technology persist despite increasing demand. Primarily, there is limited empirical evidence directly comparing digital and traditional methods for delivering post-extraction instructions for routine dental procedures like tooth extraction [1]. Moreover, few studies have explored the perspectives of both healthcare providers and patients simultaneously, a crucial step in understanding the practical implications of new technology-supported communication strategies [29,30]. Finally, the context of post-extraction care—one of the most common dental procedures that needs clear instructions—has been overlooked in digital healthcare research [19].

This study aims to address the research gap with a comprehensive cross-sectional survey comparing preferences and perceptions regarding digital versus traditional methods of delivering post-extraction instruction among dental students and patients. The specific goals are to assess preferences for digital versus traditional instruction methods among these groups; to evaluate perceptions of communication quality, confidence, and interaction; to compare these perceptions between dental students and patients; and to analyze preferences across various demographic groups.

## 2. Materials and Methods

### 2.1. Study Design and Setting

This cross-sectional survey study was conducted at the College of Dentistry, Prince Sattam bin Abdulaziz University, Al-Kharj, Saudi Arabia. The study was approved by the Standing Committee of Bioethics Research (SCBR) at Prince Sattam bin Abdulaziz University (approval number SCBR-172/2023), Al-Kharj, Saudi Arabia, and was conducted in accordance with the Declaration of Helsinki.

### 2.2. Sample Size Calculation

Sample size was calculated using the formula for estimating a single proportion (G*Power 3.1, Heinrich Heine University Düsseldorf (HHU), Germany):

*n* = (Z^2^ × *p* × (1 − *p*))/E^2^

where:

- Z = 1.96 (for 95% confidence level)
- *p* = 0.50 (expected proportion, assuming maximum variability)
- E = 0.05 (margin of error)

This calculation yielded a minimum required sample size of 384 participants. To allow separate analysis of two groups (dental students and patients) and to account for potential incomplete responses, we aimed to recruit 200 participants in each group, for a total target sample size of 400.

### 2.3. Participants and Sampling

Two distinct groups of participants were recruited using convenience sampling:

Dental Students (*n* = 200): Undergraduate dental students enrolled in clinical years (third, fourth, and fifth years) at the College of Dentistry, Prince Sattam bin Abdulaziz University, who are studying and treating patients in oral surgery and have clinical experience with patient communication regarding post-extraction care.

Inclusion criteria for students:

- Currently enrolled in clinical years (years 3–5) of the dental program
- Had clinical experience providing post-extraction instructions to patients
- Willing to participate and provide informed consent

Exclusion criteria for students:

- Students in pre-clinical years (years 1–2)
- Students on leave of absence or not actively engaged in clinical practice
- Students who declined to participate

Patients (*n* = 200): Adult patients who underwent routine tooth extraction procedures at the dental clinics of Prince Sattam bin Abdulaziz University during the study period.

Inclusion criteria for patients:

- Age ≥ 18 years
- Underwent routine single or multiple tooth extraction(s) during the study period
- Able to read and understand Arabic or English
- Owned a smartphone with QR code scanning capability

Exclusion criteria for patients:

- Age < 18 years
- Underwent complex surgical extractions needing specialized post-op care (e.g., impacted third molar surgery, cyst or tumor removal)
- Cognitive impairment or communication difficulties that would prevent completion of the questionnaire
- Did not own a smartphone or lacked QR code scanning capability
- Declined to participate

### 2.4. Post-Extraction Dental Procedures

All patients included in this study underwent routine tooth extraction procedures performed by qualified dental practitioners or supervised dental students at the College of Dentistry clinic. Routine tooth extraction refers to the removal of teeth that are visible in the mouth and can be extracted without the need for surgical flap elevation or bone removal. The procedures included:

- Simple extraction of erupted teeth due to caries, periodontal disease, or orthodontic reasons
- Extraction of mobile teeth
- Extraction of retained roots (when accessible without surgical intervention)

Standard post-extraction care instructions provided to all patients included:

Immediate post-extraction care: Bite on gauze for 30–45 min to control bleeding; avoid disturbing the extraction site.

Pain management: Take prescribed analgesics as directed; use ice packs externally for the first 24 h to reduce swelling.

Oral hygiene: Avoid rinsing or spitting vigorously for 24 h; after that, rinse gently with warm salt water 2–3 times daily; continue brushing other teeth, but avoid the extraction site for the first 24 h.

Dietary restrictions: Consume soft, cool foods during the first 24–48 h; avoid hot liquids, hard or crunchy foods, and drinking through a straw.

Activity limitations: Refrain from strenuous physical activity for 24–48 h; avoid smoking and alcohol. Warning signs: Contact your dentist if you experience excessive bleeding, severe pain unrelieved by medication, signs of infection (fever, pus, increasing swelling), or symptoms of dry socket (severe pain 3–4 days after extraction). These instructions were delivered to patients through all three methods evaluated in this study (verbal, printed leaflet, and QR code-based video), as described below.

### 2.5. Instruction Delivery Methods

All participants (both students and patients) were exposed to three different methods of delivering post- extraction care instructions:

1. Verbal Instructions: Verbal instructions were delivered by trained dental practitioners or senior dental students in a standardized manner. The verbal delivery followed a structured script covering all essential post-extraction care points and lasted approximately 3–5 min. The delivery was conducted in a quiet clinical setting, allowing for patient questions and clarification. For the purpose of this study, students observed or role-played the delivery of verbal instructions, while patients received actual verbal instructions as part of their routine post-extraction care.
2. Printed Leaflet: A printed patient information leaflet (PIL) was developed specifically for this study. The leaflet was designed in both Arabic and English, formatted on A4-size paper with the following points:

- Clear headings and subheadings for each instruction category
- Simple language appropriate for general patient literacy levels
- Bullet-point format for easy reading
- Illustrations showing proper gauze placement, ice pack application, and oral hygiene techniques
- Contact information for the dental clinic in case of emergencies

The leaflet content covered all standard post-extraction care instructions and was reviewed by three experienced oral surgery faculty members for accuracy and clarity. The leaflet was printed in color and distributed to all patients following their extraction procedures.

3. QR Code-Based Video: A QR code-based video instruction system was developed for this study. The video was professionally produced with the following specifications:

- Duration: 3 min 45 s
- Language: Arabic with English subtitles
- Content: Comprehensive coverage of all post-extraction care instructions presented by a qualified dentist
- Visual elements: Demonstration of proper techniques (gauze placement, ice pack application, gentle rinsing, soft food examples) using clinical models and animations
- Format: MP4 video file, optimized for mobile viewing

The QR code was printed on a small card (business card size) and provided to patients along with brief instructions on how to scan the code using a smartphone camera or QR code reader application. Students and patients could access the video multiple times as needed. The video was designed to be engaging, visually clear, and culturally appropriate for the Saudi Arabian context.

### 2.6. Questionnaire Development and Validation

Two separate but parallel questionnaires were developed for this study: one for dental students and one for patients. Both questionnaires were designed to assess preferences and perceptions regarding the three instruction delivery methods.

Questionnaire Domains:

Both questionnaires included the following domains:

1. Demographic information: Age, gender, educational level (for students: year of study; for patients: highest education completed), previous experience with digital health tools.
2. Delivery method preference: Participants were asked to select their preferred method (QR code-based video, printed leaflet, or verbal instructions) for various aspects of post-operative instruction delivery.
3. Perceived communication quality: Participants rated each method on its perceived effectiveness in conveying information clearly and comprehensively.
4. Confidence: Students rated their confidence in providing instructions using each method; patients rated their confidence in following instructions delivered through each method.
5. Ease of access and use: Participants rated the convenience and accessibility of each method.
6. Perceived impact on patient-provider interaction: Participants rated how each method might affect the quality of patient-provider communication and relationship.
7. Overall usefulness: Participants provided an overall rating of the usefulness of each method for post-operative care instruction delivery.

Questionnaire Format:

Questions used a combination of formats, including separate, similarly structured, validated questionnaires for each group to evaluate various aspects of each method’s effectiveness.

- Multiple-choice questions for preference selection (e.g., “Which method do you prefer for receiving/providing post-operative instructions?”)
- 3-point scales for rating perceptions (1 = disagree, 2 = Neutral, 3 = Agree)
- Ranking questions (participants ranked the three methods from 1 to 3, with 1 being most preferred)

Validation Process:

The questionnaires underwent a rigorous validation process:

1. Content validity: A panel of five experts (three oral surgery faculty members, one dental education specialist, and one health communication researcher) reviewed the questionnaires for relevance, clarity, and comprehensiveness. The Content Validity Index (CVI) was calculated, with all items achieving a CVI ≥ 0.80, indicating acceptable content validity.
2. Face validity: The questionnaires were reviewed by a separate group of 10 individuals (5 dental students and 5 patients) who were not part of the main study sample. These individuals assessed the clarity, readability, and appropriateness of the questions. Feedback was incorporated to improve the wording and formatting of questions.
3. Pilot testing: The questionnaires were pilot-tested with 30 participants (15 students and 15 patients) to assess comprehension, completion time, and identify any ambiguous items. Minor revisions were made based on pilot feedback.
4. Reliability testing: Internal consistency reliability was assessed using Cronbach’s alpha coefficient. For the student questionnaire (11 items across all domains), Cronbach’s alpha = 0.87. For the patient questionnaire (13 items across all domains), Cronbach’s alpha = 0.84. Both values indicate good internal consistency.
5. Test–retest reliability: A subset of 40 participants (20 students and 20 patients) completed the questionnaire twice, two weeks apart. Intraclass Correlation Coefficient (ICC) was calculated using a two-way mixed-effects model for absolute agreement. ICC values ranged from 0.78 to 0.92 across different questionnaire sections, indicating good to excellent test–retest reliability.

The final validated questionnaires were translated into Arabic by a certified translator and back-translated to English to ensure accuracy. Both Arabic and English versions were made available to participants based on their language preference.

### 2.7. Data Collection Procedure

Data collection followed a standardized protocol:

1. Informed consent: All participants received detailed information about the study purpose, procedures, risks, benefits, and their right to withdraw at any time. Written informed consent was obtained.
2. All participants experienced all three instruction methods in a random sequence to reduce order bias. For patients, this took place soon after their tooth extraction as part of normal post-extraction care. For students, the exposure happened during a specific study session where they reviewed all three methods related to giving post-extraction instructions.
3. Participants completed the relevant questionnaire (student or patient) after experiencing all three methods. It was given in paper form in a quiet, private environment. Research assistants were present to answer questions but avoided influencing responses. The process took approximately 10 to 15 min.
4. Data entry: Completed questionnaires were collected, checked for completeness, and entered into a secure electronic database by trained research assistants. Data entry was double-checked for accuracy.

### 2.8. Statistical Analysis

Data were analyzed using SPSS Statistics software (version 26.0, IBM Corp., Armonk, NY, USA). The analysis approach was designed to address the study objectives and account for the ordinal nature of much of the data.

Descriptive statistics: Demographic details and response distributions were summarized with frequencies and percentages for categorical data, and with means and standard deviations (SD) for continuous data. For preference questions where participants chose one of three methods, the proportion for each choice was calculated, along with 95% confidence intervals (CIs) using the Wilson score method. Normality testing: The distribution of continuous and ordinal variables was evaluated using the Shapiro–Wilk test. In many cases, non-normal distributions were observed, and non-parametric tests were used for inferential analysis.

Group comparisons: The Mann–Whitney U test was used to compare responses between dental students and patients for ordinal and continuous variables. The Mann–Whitney U test is appropriate for comparing two independent groups when the data are ordinal or when parametric assumptions are not met. Effect sizes for Mann–Whitney U tests were reported using the rank-biserial correlation coefficient (r), calculated as r = 1 − (2U)/(*n*_1_ × *n*_2_), where U is the Mann–Whitney U statistic, and *n*_1_ and *n*_2_ are the sample sizes of the two groups. Rank-biserial correlation values were interpreted as: small effect (r = 0.10–0.30), medium effect (r = 0.30–0.50), and large effect (r ≥ 0.50).

Correlation analysis: Relationships between ordinal variables (e.g., preference rankings, Likert-scale ratings) were assessed using Spearman’s rank correlation coefficient (ρ). Spearman correlation is appropriate for ordinal data and does not assume linear relationships or normal distributions. Correlation strength was interpreted as: weak (ρ = 0.10–0.30), moderate (ρ = 0.30–0.70), and strong (ρ ≥ 0.70).

Subgroup analysis: Preferences were evaluated across different demographic groups (such as age, gender, and education level) using descriptive statistics and Chi-square tests for categorical data comparisons where applicable. Statistical significance: A two-tailed alpha level of 0.05 was employed for all inferential tests. Due to the exploratory nature of some analyses and the multiple comparisons, results were interpreted with attention to both statistical significance and effect size.

## 3. Results

### 3.1. Participant Characteristics

A total of 400 participants were recruited for this study. In that, 200 were dental students and 200 patients [Table 1 and Table 2]. All 400 participants completed the questionnaires, yielding sufficient data for inclusion in the analysis (100% response rate).

Dental students: The sample included 158 males (79.0%) and 42 females (21.0%), showing statistical significance (*p* < 0.001), with a mean age of 22.73 ± 1.28 years. Students were distributed across clinical years as follows: 3rd year (*n* = 76, 38.0%), 4th year (*n* = 70, 35.0%), and 5th year (*n* = 54, 27.0%). Most students (*n* = 178, 89.0%) reported previous experience with digital tools for educational purposes.

Patients: The patient group included 110 males (55.0%) and 90 females (45.0%). The age distribution was: 18–30 years (*n* = 44, 22.0%), 31–45 years (*n* = 59, 29.5%), 46–60 years (*n* = 39, 19.5%), and over 60 years (*n* = 58, 29.0%). Educational levels were: primary education (*n* = 32, 16.0%), secondary education (*n* = 78, 39.0%), bachelor’s degree (*n* = 68, 34.0%), and postgraduate degree (*n* = 22, 11.0%). A marginally significant association was observed between education level and QR code preference (*p* = 0.050). Regarding the use of digital health tools, 134 patients (67.0%) reported using health-related mobile applications or online health information sources.

When all 400 participants were asked which method would most improve post-extraction care communication, 67.5% (*n* = 270, 95% CI: 63.2–71.6%) chose QR code-based videos, 7.5% (*n* = 30, 95% CI: 5.2–10.5%) chose printed leaflets, and 25.0% (*n* = 100, 95% CI: 21.0–29.4%) chose verbal instructions.

### 3.2. Dental Student Preferences

**Figure 1:**
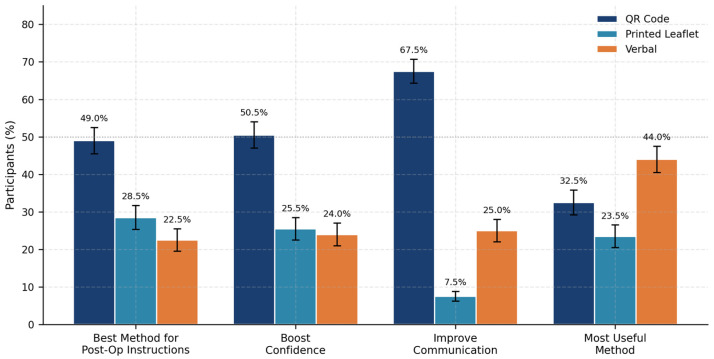
Dental Students’ Preferences for Post-extraction Instruction Delivery Methods.

Among dental students, QR code-based videos were preferred for improving patient communication (67.5%) and boosting confidence in instruction delivery (50.5%). However, verbal instructions were rated as the most useful overall (44%), followed by QR codes (32.5%) and leaflets (23.5%) [Figure 1].

### 3.3. Patient Preferences

**Figure 2:**
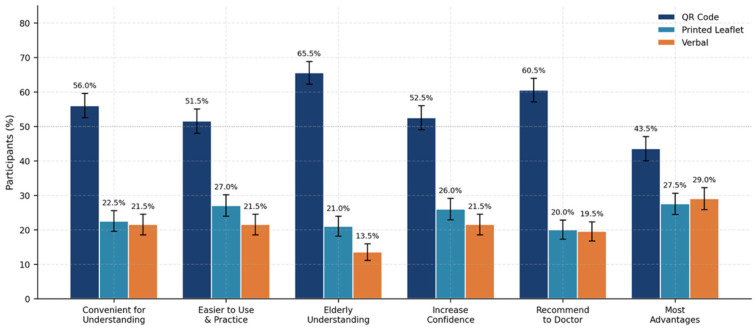
Patient Preferences for Post-extraction Instruction Delivery Methods.

Among patients, QR code-based videos were the most preferred method across all aspects presented in [Figure 2]. The highest preferences were for understanding among the elderly (65.5%) and for communicating with the dentist (60.5–recommend to the doctor).

Elderly patients aged over 60 years (*n* = 58, 29.0%) mostly preferred QR codes (65.5%) as the most convenient for understanding, while 21% preferred printed leaflets and 13.5% verbal instructions. This shows that most older adults found digital QR code methods accessible and suitable.

### 3.4. Comparison Between Dental Students and Patients

**Figure 3:**
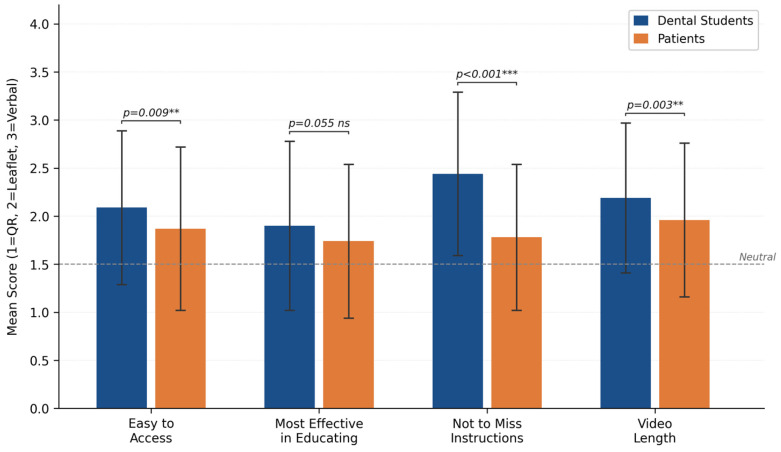
Comparison of QR Code perception score between Dental Students and Patients (Mann–Whitney U test). ** statstically significant, *** highly significant and ns—non significant.

Significant differences were found between dental students and patients across many aspects [Figure 3]. Patients rated QR code videos more favorably than dental students on ease of access (*p* = 0.009, r = −0.143), video length (*p* = 0.003, r = −0.160), and the perception that instructions wouldn’t be missed (*p* < 0.001, r = −0.446, a medium-to-large effect). No significant differences were observed for perceived educational effectiveness (*p* = 0.055).

### 3.5. Correlations Between Preference Variables

**Figure 4:**
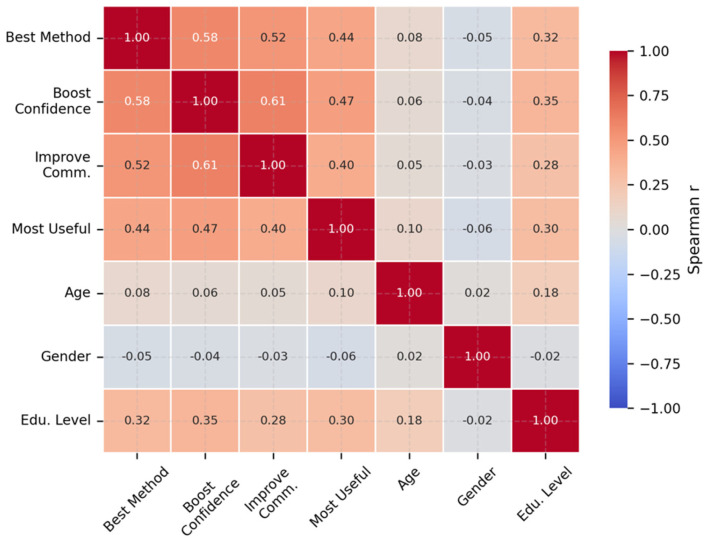
Student Group Correlation Matrix.

**Figure 5:**
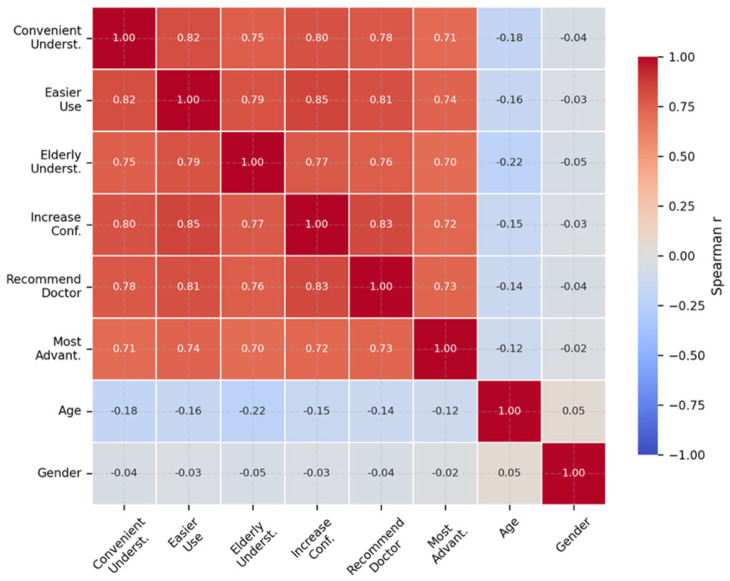
Patient Group Correlation Matrix.

Spearman’s rank correlation analysis examined relationships between preferences and perception variables [Figure 4 and Figure 5]. Key correlations include:

The Spearman correlation among dental students’ responses on different aspects of digital post-extraction instruction methods. The best method and boost confidence showed a moderate positive correlation (r = 0.58). The correlation between boosting confidence and improving communication (r = 0.61) indicates that students who pursued the digital method as the best approach also reported greater confidence and improved communication. However, other variables, such as the most useful method, demonstrated positive correlations with boost confidence (r = 0.47) and the best method (r = 0.44), suggesting favorable acceptance of the digital instruction method. Demographic characteristics, such as education level, showed weak to moderate positive correlations, with higher education exposure slightly influencing both acceptance and perceived effectiveness of digital communication. However, gender and age showed negligible or weaker correlation with the evaluated outcomes.

The Spearman correlation matrix for patients examines perceptions of digital post-extraction communication methods. Most variables show strong correlations, notably between increased confidence and ease of use (r = 0.85) and between ease of use and clear understanding (r = 0.82). This indicates that patients who received digital instructions experienced easier use, better understanding, and greater confidence in post-extraction care. Additionally, the correlation between increased confidence and willingness to recommend the doctor suggests a high likelihood of favoring digital instruction methods. Demographic factors such as age show a weak negative correlation with most variables, while gender has negligible effects. Overall, the correlation matrix highlights strong connections among usability, understanding, confidence, and satisfaction with digital postoperative instructions. These findings indicate participants who favored QR codes in one aspect generally favored them in related areas.

## 4. Discussion

This study examined preferences for digital (QR code videos) versus traditional (verbal and printed leaflet) post-extraction instructions among dental students and patients. Most participants favor QR code videos to improve patient-provider communication, with high preference rates across different aspects and demographics.

### 4.1. Interpretation of Key Findings

The perceived preference for QR code-based video instructions aligns with trends in healthcare digitalization and patient engagement [31,32]. Most dental students believe that a QR code would enhance communication and increase confidence in delivering post-extraction instructions. These results suggest that future dentists see digital tools as a valuable addition to conventional approaches. The high confidence may reflect familiarity with digital technology and recognition of multimedia’s role in enhancing information delivery [33,34].

Among patients, preferences for QR code videos differ, with the highest preference seen in the elderly. These findings challenge assumptions about older adults’ reluctance to adopt digital health technology [35,36]. The subgroup of patients aged > 60 (*n* = 58) also found QR codes the most convenient for understanding among the elderly, reflecting stereotypes and aligning with evidence that older adults are increasingly comfortable with user-friendly mobile technology when it offers clear value. Features such as replaying videos, adjusting volume, and viewing at their own pace benefit older adults, who may need more time to process health information [37,38,39].

The comparison revealed that patients favored QR codes more than students for ease of access, appropriate video length, and clarity, indicating greater perceived benefits for patients. This may be due to patients’ difficulty recalling verbal instructions during stressful postoperative periods, unlike students, who value the flexibility and immediacy of verbal communication [1,40].

While students found verbal instructions most useful compared with QR codes, they recognize the complementary benefits of both methods. Combining digital tools with traditional communication could offer the best approach, with verbal instructions addressing individual concerns and QR codes providing consistent, reusable information [31,41].

Positive correlations between preference dimensions indicate that perceptions are interconnected; those who value QR code communication improvements also report greater confidence and willingness to recommend, revealing the multifaceted benefits of digital tools [31,42].

### 4.2. Comparison with Existing Literature

This study aligns with prior research showing patients’ preferences for video-based health education over written materials [43,44]. Video instructions enhance understanding, satisfaction, and results across different healthcare environments, yet few studies compare the effectiveness of digital versus traditional methods in routine dental post-extraction care. The high acceptance of QR code instructions among older adults contrasts with earlier findings indicating that they are less receptive to digital health technology [45]. Recent evidence suggests that older adults are increasingly adopting digital tools, especially when they are easy to use and beneficial [46], and can successfully use mobile health apps with proper support. Dental students valued both digital and traditional methods, highlighting the importance of multimodal learning [47,48]. As digital technology becomes more common in dental education, students who are comfortable with these tools are more likely to incorporate them into practice [48]. The continued value of variable communication emphasizes the need to maintain strong interpersonal skills alongside technology competencies [49].

### 4.3. Implications for Practice and Education

The results of this study have significant implications for dental practice and education. The preferences for QR code-based videos suggest practices should incorporate digital tools into post-extraction care. QR codes are cost-effective and require minimal infrastructure, since most patients have smartphones [50]. Dental clinics could create standardized videos for common procedures like tooth extraction and provide QR code cards or links.

Digital tools should complement, not replace, traditional methods. Verbal instructions are important for addressing individual concerns and questions [1], while printed material can serve as a backup. Combining verbal, printed, and digital resources enhances patient understanding and satisfaction [51].

The high acceptance of QR codes among older adults indicates age isn’t a barrier to digital health tools. Practices should ensure digital tools are user-friendly, with clear instructions, and possibly demonstrate to support all patients [35].

Dental education should include training on digital communication tools, emphasizing integration with traditional methods to improve care [8,52]. Curricula should develop both technologies and interpersonal skills, vital for effective patient education [8,53].

### 4.4. Methodological Considerations

This study employed a rigorous approach, including careful sample size determination, validated questionnaires, and appropriate statistical analyses. Nonparametric tests (Mann–Whitney U test) and Spearman’s rank correlation were appropriate for the ordinal nature of the data and its non-normal distributions. Reporting effective sizes (rank biserial correlation) with *p*-value offers a fuller view of the magnitude of differences and significance.

## 5. Limitations

The study has some limitations: it assessed perceived preferences rather than actual outcomes. The design cannot establish cause-and-effect relationships, and experiencing all instruction types in a single session may have introduced order effects or bias, despite randomization. Responses might also be affected by sequence or novelty effects. Relying on self-reported data introduces biases like social desirability, and no objective measures of adherence were used. Additionally, relying on a sample from a single Saudi university restricts the generalizability, particularly considering the high smartphone usage and the mostly male demographic.

## 6. Conclusions

This study suggests that digital tools, especially QR code videos, are widely accepted by dental students and patients for post-extraction instructions. 67.5% see QR codes as better than verbal or leaflet methods, including among older adults aged ≥ 60 years (65.5%), challenging assumptions about technology barriers. However, this reflects perceptions rather than clinical effectiveness, as the study relies on self-reported preferences from a cross-sectional study. Combining verbal instructions, QR code videos, and printed leaflets could optimize communication by leveraging each method’s strengths. Further research with longitudinal designs and objective outcomes is needed to assess actual clinical benefits, cost-effectiveness, and best practices for digital tools in dentistry. The randomized clinical trials are tailored to individual patient needs and procedure types, and assess the clinical effectiveness of QR code-based instructions. While perceived acceptability is promising, evidence of improved clinical outcomes is still required. Dental practices should balance innovations with traditional methods and await further research for evidence-based adoption.

## Figures and Tables

**Table 1:** Demographic Characteristics of Dental Students (*n* = 200).

Characteristic	Category	*n*	%
Sex	Male	158	79.0
	Female	42	21.0
Year of Study	Year 3	76	38.0
	Year 4	70	35.0
	Year 5	54	27.0
Age (years)	Mean ± SD	22.73 ± 1.28	-
Smartphone Ownership	Yes	200	100.0

**Table 2:** Demographic Characteristics of patients (*n* = 200).

Characteristic	Category	*n*	%
Sex	Male	110	55.0
	Female	90	45.0
Age Group	18–30 years	44	22.0
	31–45 years	59	29.5
	46–60 years	39	19.5
	≥60 years	58	29.0
Smartphone Ownership	Yes	200	100.0

## Data Availability

The raw data supporting the conclusions of this article will be made available by the authors on request.

## Abbreviations

QR	Quick Response
PILs	Patient Information Leaflets
SCBR	Standing Committee of Bioethics Research
PSAU	Prince Sattam Bin Abdulaziz University
